# Supplementary material for: Resolving the full spectrum of human genome variation using Linked-Reads
Source: Genome Res. 2019 Apr;29(4):635–45. doi: 10.1101/gr.234443.118 (PMC6442396; doi:10.1101/gr.234443.118)
Supplement: Supplemental Material [file supp_gr.234443.118_Supplemental_longranger-2.2.zip › longranger-2.2.tar.bz2!/lr2.2-public/lib/stan/cmdstan/stan/doc/api/html/structstan_1_1math_1_1common__type_3_01_eigen_1_1_matrix_3_01_t1_00_01_r_00_01_c_01_4_00_01_eiged8accfa00e73f240c58ad02ac582ba93.html]

Stan: stan::math::common\_type< Eigen::Matrix< T1, R, C >, Eigen::Matrix< T2, R, C > > Struct Template Reference


|  |  |
| --- | --- |
|  | Stan  2.5.0  probability, sampling & optimization |


- Main Page
- Related Pages
- Namespaces
- Classes
- Files

- Class List
- Class Index
- Class Hierarchy
- Class Members

All Classes Namespaces Files Functions Variables Typedefs Enumerations Enumerator Friends Macros Pages

- stan
- math
- common\_type< Eigen::Matrix< T1, R, C >, Eigen::Matrix< T2, R, C > >

Public Types |
List of all members

stan::math::common\_type< Eigen::Matrix< T1, R, C >, Eigen::Matrix< T2, R, C > > Struct Template Reference

`#include <common_type.hpp>`

|  |  |
| --- | --- |
| Public Types | |
| typedef Eigen::Matrix  < typename common\_type< T1, T2 >  ::type, R, C > | type |
|  | |

## Detailed Description

### template<typename T1, typename T2, int R, int C> struct stan::math::common\_type< Eigen::Matrix< T1, R, C >, Eigen::Matrix< T2, R, C > >

Definition at line 23 of file common\_type.hpp.

## Member Typedef Documentation

template<typename T1 , typename T2 , int R, int C>

typedef Eigen::Matrix<typename common\_type<T1,T2>::type,R,C> stan::math::common\_type< Eigen::Matrix< T1, R, C >, Eigen::Matrix< T2, R, C > >::type

Definition at line 24 of file common\_type.hpp.

---

The documentation for this struct was generated from the following file:

- src/stan/math/matrix/common\_type.hpp


---

[ Stan Home Page ]

*© 2011–2014,
Stan Development Team.*
